# High prevalence of spotted fever group rickettsiae in ticks collected from yaks (*Bos grunniens*) in Shiqu county, eastern Tibetan Plateau, China

**DOI:** 10.3389/fmicb.2022.968793

**Published:** 2022-07-28

**Authors:** Baoshan Lin, Yin Ta, Lili Hao

**Affiliations:** ^1^College of Animal Husbandry and Veterinary Medicine, Southwest University for Nationalities, Chengdu, China; ^2^Animal Disease Prevention and Control Center of Aba Tibetan and Qiang Autonomous Prefecture, Markang, China

**Keywords:** SFG rickettsiae, ticks, Shiqu, high prevalence, yaks (*Bos grunniens*)

## Abstract

Tick-borne diseases have become a global health concern in recent decades. Spotted fever group (SFG) rickettsiae have been recognized as important pathogens of human tick-borne diseases worldwide. In this study, *Dermacentor everestianus* (*n* = 646) and *Haemaphysalis qinghaiensis* (*n* = 172) ticks were collected from yaks (*Bos grunniens*) in Shiqu county, eastern Tibetan Plateau, China. SFG rickettsiae were identified and characterized in these ticks. A total of 49.9% (408/818) ticks were infected by *Rickettsia* spp. with infection rates of 58.1% (100/172) and 46.7% (308/646) detected in *H. qinghaiensis* and *D. everestianus* ticks, respectively. Furthermore, 95% of *Rickettsia* spp. were *Rickettsia raoultii*-like bacteria, and 5% were related to *Candidatus* Rickettsia longicornii. To the best of our knowledge, this is the first time that SFG rickettsiae infections were firstly reported in Shiqu county for these tick species. Our results indicated that *H. qinghaiensis* and *D. everestianus* ticks from Shiqu county became highly infected with a *R. raoultii*-like bacteria during their feeding process. This observation is alarming because of the zoonotic potentiality of these species. Overall, the present study detected a widespread of *R. raoultii*-like bacteria in ticks that are considered a serious threat to domestic animals and humans in Shiqu county. The prevalence of *R. raoultii*-like bacteria in human and wildlife hosts should be further investigated in the future.

## Introduction

Rickettsioses are important emerging or reemerging vector-borne diseases that are distributed worldwide and have increasingly challenged public health services in recent years. The significance of ticks (Acari: Ixodida) has long been recognized due to their ability to feed on a variety of host species and transmit *rickettsial* pathogens that can infect various vertebrate hosts, including humans. Members of the genus *Rickettsia* have been classified into five major groups, including the spotted fever group (SFG), the typhus group (TG), the transitional group (TRG), the *Rickettsia bellii* group and the *Rickettsia canadensis* group (Weinert et al., 2009; Merhej et al., 2014). In China, many SFG rickettsiae belong to *Rickettsia sibirica* group, including: (1) *R. sibirica* subsp. *sibirica*, a North Asian tick typhus pathogen, detected in *Dermacentor silvarum* and *Dermacentor sinicus* found in northern China (Yu et al., 1993); and (2) *R. sibirica* subsp. *mongolitimonae*, a lymphangitis-associated rickettsiosis pathogen, isolated from *Hyalomma asiaticum* from Inner Mongolia (Yu et al., 1993). Most recently, Zhang et al. (2006) have reported that the *Rickettsia* identified in *Haemaphysalis flava* from Cangxi county, Sichuan province, China, was most closely related to a member of the *Candidatus* Rickettsia gannanii subgroup identified in *Haemaphysalis qinghaiensis* (Yang et al., 2016). Other *Rickettsia* species causing SFG rickettsiosis have been discovered in China, including *Rickettsia heilongjiangensis, R. sibirica, Rickettsia raoultii, Rickettsia slovaca, Rickettsia felis, Rickettsia aeschlimannii*, and *Rickettsia massiliae* (Wei et al., 2015; Guo et al., 2016).

However, there are very few reports on the occurrence of SFG rickettsiae and their vectors in the eastern Tibetan Plateau especially in Shiqu county (average altitude of 4,200 m above sea level) (Wang et al., 2012). In Shiqu, yaks (*Bos grunniens*) are the largest population of local livestock (around 600,000 individuals) and are the main source of income for local residents, producing butter, milk and meat. Due to their traditional lifestyle, yaks live in close contact with local residents (especially yak farmers), and severe tick infestation has often been observed. Therefore, in this study, we aim to investigate the prevalence of *Rickettsia* spp. in ticks collected from yaks in Shiqu county and identify the degree of spread of these pathogens and their impact on livestock in the region, which would provide preliminary data for further human infection risk research.

## Materials and methods

### Sample collection and molecular identification

**FIGURE 1:**
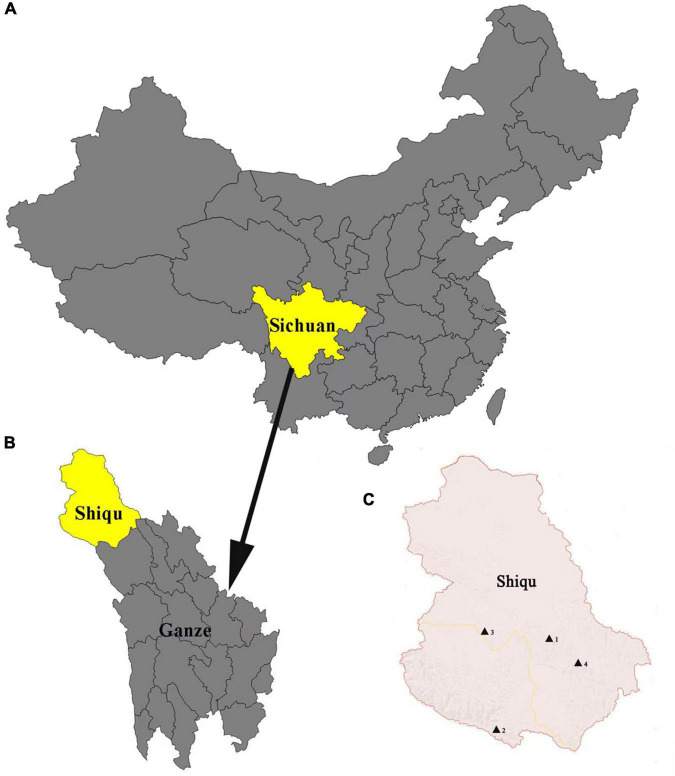
Map of Shiqu county. **(A)** Map of China where Sichuan province is marked in yellow. **(B)** Map of Ganze, Tibetan autonomous prefecture, where Shiqu county is marked in yellow. **(C)** Map of Shiqu country, where the four village locations in this study (1. Ariza, 2. Maga, 3. Derongma, and 4. Changxgma) are marked by black triangles.

Ticks were collected from yaks in Derongma, Changxuganma, Arizha, and Maga villages in Shiqu county (35°58′ 50.77′′N, 98°06′ 10.58′′E) (Figure 1) from June to August 2018. Ticks were stored in 70% ethanol at 4°C. Approximately 5–6 ticks were collected from each yak. All tick samples were identified by morphological characteristics with standard taxonomic keys (Deng and Jiang, 1991) and further confirmed using PCR amplification targeting the small subunit 16S rRNA gene (Black and Piesman, 1994). Primer sequences are listed in Supplementary Table 1. Detailed information regarding tick collection is listed in Supplementary Table 2. In addition, 120 blood samples were collected from slaughtered yaks in an abattoir in November 2018.

### DNA extraction and PCR amplification

All ticks were sectioned longitudinally and processed individually for DNA extraction using a TIANamp Genomic DNA Kit (TIANGEN Biotech Co., Ltd., Beijing, China, Cat. No. DP304) according to the manufacturer’s protocol. DNA of all blood samples was extracted with a Blood Genomic DNA Kit (Transgen Biotech Co., Ltd., Beijing, China, Cat. No. EE121-11) and stored at −20°C until analysis. PCR amplification was performed targeting the *ompA* (Oteo et al., 2006) and *ompB* genes (Fernandez de Mera et al., 2013) according to previously published criteria (Raoult et al., 2005). Primer sequences are listed in Supplementary Table 1. PCR was performed in a total reaction volume of 25 μl, including 2 μl template DNA, 12.5 μl 2 × PCR mix (TransGen Biotech Co., Ltd., Beijing, China, Cat. No. AS111), and 20 pmol of each primer (Sangon Biotech Co., Ltd., Shanghai, China). After an initial denaturation for 3 min at 95°C, 40 cycles of denaturation for 30 s at 94°C, annealing for 30 s at 55°C and elongation for 30 s at 72°C were performed, followed by a final extension step at 72°C for 7 min. The final amplification products were sent for sequencing (Sangon Biotech Co., Ltd., Shanghai, China).

### Bioinformatics/phylogenetic analysis

Firstly, the obtained sequences were analyzed and manually edited using the BioEdit program (Version 7.0.9). Then sequences were compared to each other using DNASTAR v.7.1.0. Subsequently, in order to assign them identities, each sequence underwent a homology comparison using the previously described BLAST tool in order to compare with sequences deposited in GenBank (Altschul et al., 1990; Benson et al., 2002). Phylogenetic trees were constructed with Neighbor-Joining using MEGA 6 software based on the *ompA* and *ompB* genes, respectively. The evolutionary distance was computed using Kimura 2-parameter method with 1,000 bootstrap replicates. The phylogenetic tree was drawn to scale and branch lengths were measured as the number of substitutions per site.

### Statistics

A Pearson Chi-square test using SPSS (ver. 19.0, IBM, New York, NY, United States) was applied to compare the prevalence of *Rickettsia* spp. among the different sampling locations and tick species. Results with *P* < 0.05 were considered significant.

## Results

In total, 818 adult ticks (Changxuganma, *n* = 234; Maga, *n* = 224; Derongma, *n* = 192; and Arizha, *n* = 168) were collected and identified. Molecular identification of ticks based on the mitochondrial subunit 16S rRNA gene confirmed the presence of two different tick species belonging to *Dermacentor everestianus* (*n* = 646) and *H. qinghaiensis* (*n* = 172). Ticks were first screened using the *Rickettsia* spp. *ompA* gene. *ompA*-positive samples were then screened for the *ompB* gene. According to the defined criteria for *Rickettsia* species, 49.9% of the samples (408/818) were identified as SFG rickettsiae (Table 1). However, all yak blood samples were negative.

**TABLE 1:** Prevalence of *Rickettsia* spp. in ticks collected from yaks in Shiqu county.

Location	No. of samples		Infection rate %		
		
	*H. qinghaiensis*	*D. everestianus*	*H. qinghaiensis*	*D. everestianus*	Total
Ariza	0/168	168/168	0	42.8 (72/168)	42.8
Maga	172/224	52/224	58.1 (100/172)*	38.4 (20/52)	53.5
Derongma	0/192	192/192	0	58.3 (112/192)	58.3**
Changxgma	0/234	234/234	0	44.4 (104/234)	44.4
Total	172	646	58.1 (100/172)	47.7 (308/646)	49.9

**Derongma: Ariza/Changxgma, P < 0.01.

*Maga: H. qinghaiensis/D. everestianus, P < 0.01.

**FIGURE 2:**
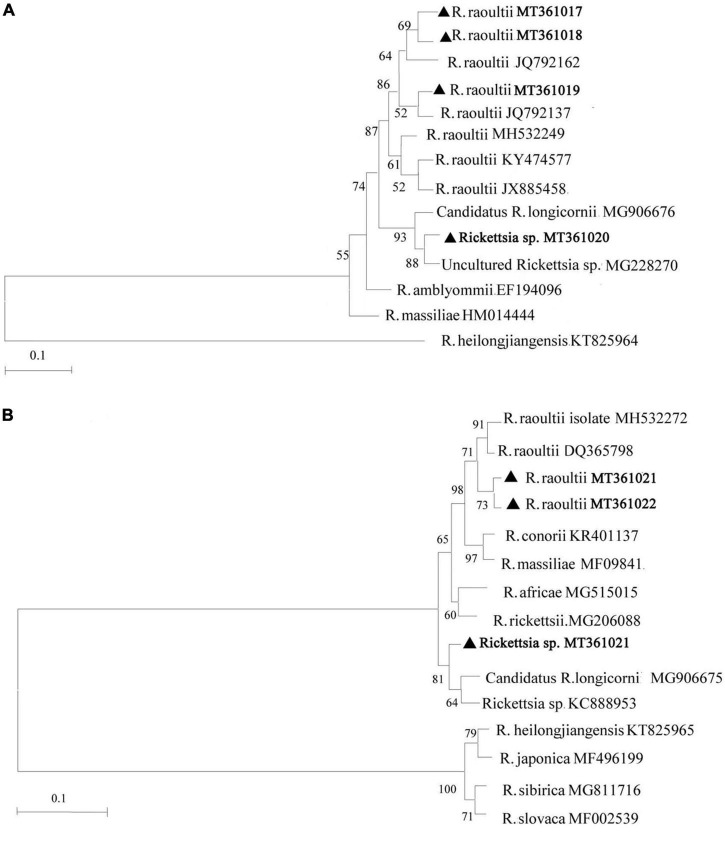
Phylogenetic analysis of *Rickettsia* spp. identified in Shiqu county. Neighbor-joining (NJ) phylogenetic trees were constructed based on *Rickettsia ompA* **(A)** and *ompB* **(B)**, respectively. The sequences obtained in this study are marked with black triangles.

Based on the analysis of *ompA* and *ompB* amplicons, we discovered 7 unique sequences from the 408 positively identified samples and they were submitted to GenBank with accession numbers as follows: MT361017, MT361018, MT361019, and MT361020 for the *ompA* gene; MT361021, MT361022, and MT361023 for the *ompB* gene. Sequence analysis of the *ompA* gene indicated that the MT361017, MT361018, and MT361019 sequences were closely related to *R. raoultii* (JQ792162 and JQ792137) with a sequence identity of 99.8–100%. Sequence MT361020 was 98.2–100% identical to *Candidatus* R. longicornii (MG906676) and an uncultured *Rickettsia* sp. isolated from Korea and Qinghai (MG228270), respectively. For the *ompB* gene, sequences MT361021 and MT361022 were 99.3–99.5% identical to *R. raoultii* (MH532272 and DQ365798). Sequence MT361023 showed 100% identity to *Rickettsia* sp. (KC888953) and *Candidatus* R. longicornii (MG906675) isolated from Korea. Furthermore, according to defined threshold identity for the SFG rickettsiae species (*ompA* ≥ 98.8% and *ompB* ≥ 99.2%) (Raoult et al., 2005), *ompA* and *ompB*-based phylogenetic analysis supported the classification and detection of *Rickettsia* spp. in this study (Figure 2). The sequences are available in the Supplementary Material.

*Rickettsia raoultii*-like bacteria were positively identified in 95% of cases, and *Rickettsia* spp., accounting for 5% of cases was only detected in *H. qinghaiensis* ticks. The infection rate of *R. raoultii*-like bacteria in Derongma village was 58.3%, significantly higher than in Ariza and Changxuganma villages (CI = 95%, *P* < 0.01, Table 1). In Maga village, the infection rate (58.1%) in *H. qinghaiensis* was significantly higher than *D. everestianus* (38.4%; CI = 95%, *P* < 0.01, Table 1). Overall tick infection rates in Shiqu county with *Rickettsia* spp. were 58.1% in *H. qinghaiensis* and 46.7% in *D. everestianus*, respectively.

## Discussion

Two tick species (*H. qinghaiensis* and *D. everestianus*) infesting yaks from Shiqu county were identified. *H. qinghaiensis* was only identified in yak from Maga village, whereas *D. everestianus* has earlier mainly been found in all four villages. Previously *D. everestianus* was only reported in northwestern China and Nepal (Chen et al., 2014) at an altitude of 2,600–4,700 m (Apanaskevich et al., 2014). Larvae and nymphs from this tick species often infect lagomorphs and rodents, while adult ticks usually infect medium-size domestic and wild mammals such as hare (*Lepus oiostolus*), sheep, yaks, and horses (Apanaskevich et al., 2014; Chen et al., 2014). However, *H. qinghaiensis* has only been recorded in China (Gao et al., 2007a,b, 2008, 2009; Li et al., 2007), and is particularly prevalent in Qinghai, Gansu, Sichuan, and Tibet provinces (Weinert et al., 2009). *H. qinghaiensis* are usually more active at low than high altitudes. In this study, Arizha, Changxuganma, and Derongma villages belong to a sub-frigid zone, with an altitude between 4,300–4,600 m, while Maga village is located in a cold temperate zone, with an altitude of 3,799 m. There are significant differences in altitude between Maga and the other three villages, which is probably the reason *H. qinghaiensis* was only found in Maga.

*Rickettsia raoultii* was first isolated from ticks in Russia in 1999 (Rydkina et al., 1999) and has been found mainly in *Dermacentor* spp. ticks in several countries in Europe (Speck et al., 2012; Foldvari et al., 2013). Human infections of *R. raoultii* have been reported in recent years. In 2012, *R. raoultii* DNA was detected in two patients from Mudanjiang, China (Jia et al., 2014). In 2015 and 2016, 26 cases of humans infected of *R. raoultii* were reported in Inner Mongolia (Jia et al., 2014; Li et al., 2018), and in Henan and Shandong provinces, China (Li et al., 2018). The Khabarovsk *R. raoultii* strain was isolated from the blood of a patient (Li et al., 2018), which showed high genetic identity with the *R. raoultii*-like bacteria identified in our study.

*Rickettsia raoultii* has been found in at least 26 tick species belonging to 7 genera, including *Dermacentor* (Jia et al., 2014), *Haemaphysalis* (Li et al., 2018; Zheng et al., 2018), *Amblyomma* (Parola et al., 2013), *Ixodes* (Shpynov et al., 2009; Rar et al., 2017), and *Hyalomma* (Yin et al., 2018), although *Dermacentor* spp. ticks are the main vector (Mediannikov et al., 2008; Wang et al., 2012). In China, *R. raoultii*-like bacteria were first detected in Tibet (Wang et al., 2012). In this study, infection rates of *R. raoultii*-like bacteria in *D. everestianus* and *H. qinghaiensis* were 47.6 and 58.1%, respectively, suggesting that these tick species may be possible vectors for *R. raoultii* in Shiqu county, although *Haemaphysalis* species including *Haemaphysalis longicornis*, *Haemaphysalis erinacei*, and *Haemaphysalis verticalis*, may also be efficient vectors of *R. raoultii*.

Our study did not find *Rickettsia* spp. DNA in yak blood samples (*n* = 120). Because of local religious customs regarding yaks, it was impossible to obtain blood samples from live animals without the permission of their owners. Instead, all blood samples were collected from slaughtered yaks in an abattoir in November 2018 without *Rickettsia* spp. DNA detected. In order to obtain more detailed prevalence information of *Rickettsia* spp. in ticks, tick samples should be collected from other hosts, including sheep, horse, rodents, and plateau pika (*Ochotona curzoniae*). It would be much more important to collect unfed questing ticks from the vegetation and investigate their infection rates. In Shiqu county, there are about 1.3 billion plateau pikas and other rodents (*Eospalax fontanierii*, *Lasiopodomys fuscus*, etc.), which represent the largest mammalian populations in the area. These small mammals are natural hosts of *D. everestianus* larvae and nymphs and might play important roles in the natural transmission cycle and dispersal of *Rickettsia* spp. Future studies could also investigate *Rickettsia* spp. in spleen and/or blood samples from these small mammals. Also, it would be much more important to investigate their reservoir competence for the involved *Rickettsia* species. Alternatively, seasonal infection risk for human and animals could be estimated by dragging blankets across grasslands to capture and calculate the numbers of unfed ticks in a given area. At present, no reports were found of SFG rickettsiae (identified in *H. qinghaiensis* and *D. everestianus*) linked to human cases from the Tibet Plateau. Due to their traditional lifestyle, local people (and particularly herdsmen) do not tend to go to hospital unless they have serious medical issues, resulting in very few reported or recorded clinical cases. However, in the future, we should pay close attention to human tick bite cases in Shiqu.

## Conclusion

In summary, we have shown, for the first time high prevalences of *Rickettsia* spp. in *D. everestianus* and *H. qinghaiensis* ticks sampled from yaks in Shiqu county. In this region, key mammalian tick hosts are domesticated yak and wild mammals such as rodents and plateau pika. A more comprehensive study of *Rickettsial* pathogens to further assess the prevalence of *Rickettsia* spp. in other livestock and wildlife hosts from Shiqu county should be made in the future.

## Data availability statement

The datasets presented in this study can be found in online repositories. The names of the repository/repositories and accession number(s) can be found below: https://www.ncbi.nlm.nih.gov/genbank/, MT361017, MT361018, MT361019, MT361020, MT361021, MT361022, and MT361023.

## Author contributions

LH conceptualized and designed the experiments and wrote the manuscript. BL and YT performed the experiments and analyzed the data. BL analyzed the data. All authors have reviewed and agreed with the manuscript.

## Supplementary material

Supplementary Table 1 Primer sequences used for tick and *Rickettsia* spp. identification.

Supplementary Table 2 Tick sampling information.

Supplementary Text 1 *ompA* gene sequence.

Supplementary Text 2 *ompB* gene sequence.
