# Supplementary material for: High prevalence of spotted fever group rickettsiae in ticks collected from yaks (Bos grunniens) in Shiqu county, eastern Tibetan Plateau, China
Source: Front Microbiol. 2022 Jul 28;13:968793. doi: 10.3389/fmicb.2022.968793 (PMC9366146; doi:10.3389/fmicb.2022.968793)
Supplement: Supplementary Table 1 — Primer sequences used for tick and Rickettsia spp. identification. [file Table_1.DOCX]

**Supplementary Table 1.** Primer sequences used for tick and *Rickettsia* spp. identification.

| Target gene | Primer sequence（5'-3'） | Product (bp) | References |
| --- | --- | --- | --- |
| 16SrRNA | 16S+1: CTGCTCAATGATTTTTTAAATTGCGG  16S-1: CCGGTCTGACAGATCAAGT | 460 | Black, *et al*., 1994 |
| ompA  ompB | Rr190.70p: ATGGCGAATATTTCTCCAAAA | 530 | Oteo, *et al.*, 2006 |
|  | Rr190.602n: AGTGCAGCATTCGCTCCCCCT  OmpB-F: GGGTGCTGCTACACAGCAGAA  OmpB-R: CCGTCACCGATATTAATTGCC | 618 | Fernandez, *et al*., 2013 |
