# Supplementary material for: High prevalence of spotted fever group rickettsiae in ticks collected from yaks (Bos grunniens) in Shiqu county, eastern Tibetan Plateau, China
Source: Front Microbiol. 2022 Jul 28;13:968793. doi: 10.3389/fmicb.2022.968793 (PMC9366146; doi:10.3389/fmicb.2022.968793)
Supplement: Supplementary Table 2 — Tick sampling information. [file Table_2.DOCX]

**Supplementary Table 2.** Tick sampling information.

| Collection site | No. of yaks | Tick species |  | Stage | No. of ticks |
| --- | --- | --- | --- | --- | --- |
| Ariza | 28 | *D. everestianus* |  | Female  Male | 72  96 |
| Maga  Derongma  Changxgma | 44  11  32  39 | *H. qinghaiensis*  *D. everestianus*  *D. everestianus*  *D. everestianus* |  | Female  Male  Nymph  Female  Male  Female  Male  Female  Male  Nymph | 103  62  7  29  23  84  108  135  95  4 |
