# Supplementary material for: High prevalence of spotted fever group rickettsiae in ticks collected from yaks (Bos grunniens) in Shiqu county, eastern Tibetan Plateau, China
Source: Front Microbiol. 2022 Jul 28;13:968793. doi: 10.3389/fmicb.2022.968793 (PMC9366146; doi:10.3389/fmicb.2022.968793)
Supplement: Supplementary Text 1 — ompA gene sequence. [file Data_Sheet_1.DOCX]

>R.raoultii MT361017

CCAAAATTATTTCAAAAAGCAATACAACAAGGTCTTAAAGCCGCTTTATTCACCACCTCAACCGCAGCGATAATGCTGAG

TAGTAGCGGGGCACTCGGTGTTGCTGCAGGTGTTATTTCTACTAATAATGCAGCATTTAATGATCTTGCTGTTGCCAATA

ATTGGAATGAGATAACGGCTGGAGGGGTAGCTAATGGTACTCCTGCTGGTGGTCCTCAAGACAATGGGGCATTTACTTAC

GGTGGTGATCATACTATCACTGCAGATGAAGCAGGTCGTATTATTACAGCTATAAATGTTGCGGGTACTACTCCTGTAGG

TCTAAATATTACTCAAAATACCGTCGTTGGTTCGATTTTGACGGGAGGTAACTTGTTGCCTGTTACTATTACTGCCGGCA

AAAGCTTAACTTTAAACGGTACTAATGCTGTTGCTGCAAATCATGGTTTTGATGCTCCTGTCGATAATTATACAGGTTTA

GGAAATATAACTTTAGGGGGA

>R.raoultii MT361018

AAAATTATTTCAAAAAGCAATACAACAAGGTCTTAAAGCCGCTTTATTCACCACCTCAACCGCAGCGATAATGCTGAGTA

GTAGCGGGGCACTCGGTGTTGCTGCAGGTGTTATTTCTACTAATAATGCAGCATTTAATGATCTTGCTGTTGCCAATAAT

TGGAATGAGATAACGGCTGGAGGGGTAGCTAATGGTACTCCTGCTGGTGGTCCTCAAGACAATGGGGCATTTACTTACGG

TGGTGATCATACTATCACTGCAGATGAAGCAGGTCGTATTATTACAGCTATAAATGTTGCGGGTACTACTCCTGTAGGTC

TAAATATTACTCAAAATACCGTCGTTGGTTCGATTGTGACGGGAGGTAACTTGTTGCCTGTTACTATTACTGCCGGCAAA

AGCTTAACTTTAAACGGTACTAATGCTGTTGCTGCAAATCATGGTTTTGATGCTCCTGCCGATAATTATACAGGTTTAGG

AAATATAACTTTAGGGGGA

>R.raoultii MT361019

CAAAATTATTTCAAAAAGCAATACAACAAGGTCTTAAAGCCGCTTTATTCACCACCTCAACCGCAGCGATAATGCTGAGT

AGTAGCGGGGCACTCGGTGTTGCTGCAGGTGTTATTTCTACTAATAATGCAGCATTTAATGATCTTGCTGTTGCCAATAA

TTGGAATGAGATAACGGCTGGAGGGGTAGCTAATGGTACTCCTGCTGGTGGTCCTCAAGACAATGGGGCATTTACTTACG

GTGGTGATCATACTATCACTGCAGATGAAGCAGGTCGTATTATTACAGCTATAAATGTTGCGGGTACTACTCCTGTAGGT

CTAAATATTACTCAAAATACCGTCGTTGGTTCGATTTTGACGGGAGGTAACTTGTTGCCTGTTACTATTACTGCCGGCAA

AAGCTTAACTTTAAACGGTACTAATGCTGTTGCTGCAAATCATGGTTTTGATGCTCCTGCCGATAATTATACAGGTTTAG

GAAATATAACTTTAGGGGGA

>Rickettsia sp. MT361020

TTATTTCAAAAAGCAATACAACAAGGTCTTAAAGCCGCTTTATTCACCACCTCAACCGCAGCGATAATGCTGAGTAGTAG

TGGGGTACTCGGTGTTGCTGCAGGTGTTATTGCTACTAATAATGATGCAGCATTTAGTAATAATGCTGCTGCCAATAATT

GGAATGAGATAACGGCTGGAGGGGTAGCTAATGGTATTCCTGCTGGCGGTCCTCAAAACAATTGGGCATTTACTTACGGT

GGTGATTATACTATCACTGCAGATGTAGTCGATCGTATTATTACGGCTATAAATGTTGCGGGTACTACTCCCGTAGGTCT

AAATATTGCTCAAAATACCGTCGTTGGTTCGATTATAACTGGAGGTAACTTGTTGCCTGTTACTATTGCTGGCAAAAGCT

TAACTTTAAACGGTACTAATGCTGTTGCTGCAAATCATGGTTTTGATGCTCCGGCCGATAATTATACAGGTTTAGGAAAT

ATAACTTT
