# Supplementary material for: High prevalence of spotted fever group rickettsiae in ticks collected from yaks (Bos grunniens) in Shiqu county, eastern Tibetan Plateau, China
Source: Front Microbiol. 2022 Jul 28;13:968793. doi: 10.3389/fmicb.2022.968793 (PMC9366146; doi:10.3389/fmicb.2022.968793)
Supplement: Supplementary Text 2 — ompB gene sequence. [file Data_Sheet_2.DOCX]

>R.raoultii MT361021

CAACAAACGGAGCTGCTACAACTGTTGATGGTGCGGGATTTGACCAAACTGCCGCTCCTGCAAATGTTGCGGTTGCTCTA

AATGCAGTTATTACTGCTAATGCTAATAATGGTATTAATTTAAATACTCCAGCCGGTAGTTTTAACGGTTTGTTTTTAGA

TACTGCACACAATTTAGCAGTGACAGTGAGTGCAGATACTACCTTAGGGTTCATCACTAATGCTGCTAATAACGGTAACT

CCTTTAACCTTACGCTTGGTGCCGGTAAAACTCTTACTATAACAGGTCAAGGTATTACTAATGCACAAGCTGCTGTTACA

AAAAATGCTCGAAATGTTGTTGCACAATTTAATGGTGGTGCTGCTATTGCCAATAATGATCTTAGCGGTGTAGGAACAAT

AGACTTGGGTGCTGCGGCTTCTACATTAGTATTTAATTTAGCAAATCCTACAACTCAAAAAGCTCCTCTTATACTTGGAA

ATAATGCTGTAATAGCTAATGGTGTTAACGGTACATTAAATGTTACTAATGGATTTATTCAAGTTTCAGATAAAAGTTTT

GCTACTGTTAA

>R.raoultii MT361022

TAGAACAACAAAGGGAGCTGCTACAACTGTTGATGGTGCGGGATTTGACCAAACTGCCGCTCCTGCAAATGTTGCGGTTG

CTCTAAATGCAGTTATTACTGCTAATGCTAATAATGGTATTAATTTAAATACTCCAGCCGGTAGTTTTAACGGTTTGTTT

TTAGATACTGCACACAATTTAGCAGTGACAGTGAGTGCAGATACTACCTTAGGGTTCATCACTAAGGCTGCTAATAACGG

TAACTCCTTTAACCTTACGCTTGGTGCCGGTAAAACTCTTACTATAACAGGTCAAGGTATTACTAATGCACAAGCTGCTG

TTACAAAAAATGCTCGAAATGTTGTTGCACAATTTAATGGTGGTGCTGCTATTGCCAATAATGATCTTAGCGGTGTAGGA

ACAATAGACTTGGGTGCTGCGGCTTCTACATTAGTATTTAATTTAGCAAATCCTACAACTCAAAAAGCTCCTCTTATACT

TGGAAATAATGCTGTAATAGCTAATGGTGTTAACGGTACATTAAATGTTACTAATGGATTTATTCAAGTTTCAGATAAAA

GTTTTGCTACTGTTAA

>Rickettsia sp. MT361023

TAGAACAACAAACGGAGTTGCTACAACTGTTGATGGTGCGGGATTTGACCAAACTGTCAATCTTGCAAATGTTGCAGTTG

CTCTAAATGCAGTTATTACTGCTAATGTTAATAATGGTATTAATTTCAATACTCCAGCCGGTAGTTTTAACGGTTTGTTT

TTAAATAATGCAAACCATTTAGCAGTGACAGTGAGTGAAGATACTACCTTAGGGTTCATCAATAATGTTGCTCATAACGC

TAACTTCTTTAACATTACGCTTGATGCCGGTAAAACTCTTACTATAACAGGTCAAGGTATTACTAATGTACAAGCTGCTG

CTACACACAATGCTCAAAATATTGTTGCACAATTTAATGGTGGTGCTGCTATTGCCAATAATGATCTTAGCGGTGTAGGA

ACAATAGACTTCGGTGCTGCGGCTTCTACATTAGTATTTAATTTAGCAAATCCTACAACTCAAAAAGCTCCTCTTATACT

TGCAGGTAATGCTTTAATAGCTAATGGTGCTAACGGTACATTAAATGTTACTAATGGATTTATTCAAGTTTCAGATAAAA

GTTTTGCTACTGTTAA
